# The unbearable lightness of laughting: a reflexive thematic analysis of smiles and laughter in five psychotherapy training processes

**DOI:** 10.3389/fpsyg.2025.1720110

**Published:** 2025-11-18

**Authors:** Cecilie Hillestad Hoff, Hanne Strømme

**Affiliations:** Department of Psychology, University of Oslo, Oslo, Norway

**Keywords:** containment, nonverbal communication, psychoanalytic interpretations, psychotherapy training, smiles and laughter in psychotherapy, supervision, qualitative analysis

## Abstract

**Objective:**

This study explored how smiles and laughter unfolded in five psychotherapy training processes, comprising two psychodynamic, two metacognitive, and one integrative.

**Methods:**

Using a multimodal approach, video observations from naturalistic therapy and supervision sessions served as a springboard for Interpersonal Process Recall interviews with therapists, clients, and supervisors. Transcripts from supervision sessions and interviews were analyzed with Reflexive Thematic Analysis.

**Findings:**

The analysis yielded four themes: 1. Smiles and laughter sometimes served to strengthen the therapeutic alliance, while at other times they functioned as emotion-regulating strategies or carried profound personal significance; 2. The therapists intuitively tended to downregulate their responses to clients’ expressions of laughter, to modulate and contain the clients’ underlying emotions; 3. The way therapists handled laughter and smiles in the therapeutic setting seemed to be related to their degree of security and the quality of the therapeutic relationship; and 4. In supervision, smiles and laughter were not explicitly addressed as a distinct theme but occasionally surfaced spontaneously during sessions.

**Conclusion:**

By showing how clinical practice unfolds on observable and inferred emotional levels, the study highlights the importance of empirical grounding and the difficulty of verbalizing subtle nonverbal processes.

## Introduction

In psychotherapy research, therapeutic competence has traditionally been explored through verbal relational skills. Yet, a growing body of research highlights the essential role of embodied and nonverbal processes in effective therapeutic practice (Atzil-Slonim et al., 2023; Bar-Kalifa et al., 2023; Deres-Cohen et al., 2021; Hill et al., 2025; Norcross and Wampold, 2011; Zilcha-Mano, 2024). In a previous paper, we defined *nonverbal relational competence* as the therapist’s ability to perceive and respond sensitively to nonverbal expressions and interactional patterns, while simultaneously regulating their own bodily signals and using this awareness to guide clinical interventions that promote therapeutic change (present authors, submitted study).

Within psychoanalysis, nonverbal competence has long been regarded as foundational to emotional attunement and containment (e.g., Bion, 1962; Freud, 1923; Winnicott, 1971). However, such competence is rarely examined at the level of observable behavior. For example, in Tuckett’s (2005) seminal paper “Does Anything Go?,” the topic is addressed only implicitly, referring shortly to “what is sensed,” before quickly moving on to theoretical interpretation. This tendency to bypass the descriptive level raises important questions about how clinicians perceive, reflect upon, and make use of nonverbal cues in real therapeutic interactions.

In this study, we examine in detail the expressions of smiles and laughter in psychotherapy training. Although smiles and laughter in psychotherapy remain underexplored, some empirical studies have examined them in clinical settings, underscoring their complexity. Studies by Benecke and Krause (2005) and Dreher et al. (2001) show that therapist smiling and mimicry are not inherently beneficial; rather, therapeutic progress depends on the therapist’s selective, emotionally attuned modulation of nonverbal responses. Excessive mirroring may hinder outcomes, while affective incongruence can undermine the therapeutic alliance. Other research has shown that shared laughter may strengthen the therapeutic bond (Bedi et al., 2005; Darwiche et al., 2008; Marci et al., 2004; Ramseyer and Tschacher, 2011; Seikkula et al., 2015), while also serving other interpersonal functions such as regulating affect (Koole, 2009), expressing disagreement with the therapist (Canestrari and Dionigi, 2018), managing distance, or masking vulnerability (Hill et al., 2025; Pomeroy and Weatherall, 2014; Bänninger-Huber and Salvenauer, 2022). Hill et al. (2025) found that clients with avoidant attachment styles tended to laugh more frequently as a distancing strategy, whereas anxiously attached clients laughed less, and typically in distress related contexts.

Beyond the clinical field, interdisciplinary research has developed a rich understanding of nonverbal phenomena in everyday interaction, which contributes significantly to our understanding of these expressions in clinical settings. Developmental and infant research, for example, has made major contributions to understanding these dynamics (e.g., Beebe et al., 2010; Feldman et al., 1996; Tronick and Beeghly, 2011). Pioneers such as Beebe and Lachmann (2002) demonstrated that the micro-coordination of gaze, gesture, and affect between infant and caregiver forms the foundation for later relational regulation. These insights have profoundly influenced psychotherapy theory, suggesting that therapeutic relationships may rely on similar moment-to-moment processes of affective attunement and repair. Moreover, conversation analytic and sociolinguistic studies (e.g., Glenn, 2003; Holt, 2016; Haakana, 2012) support that laughter functions not only as a marker of joy but as a nuanced communicative act, managing tension, aligning participants, or regulating intimacy and distance. Huron (2006), drawing on music psychology and affective neuroscience, proposes that laughter can arise from a violated expectation that is rapidly resolved as harmless, producing a physiological response of relief. This “relief laughter” highlights how seemingly simple expressions can emerge from complex emotional and cognitive processes.

Emotion theory also complicates any simplistic reading of these expressions. While Ekman’s (Ekman and Friesen, 1982; Ekman, 1992) categorical theory posits a small set of biologically hardwired universal emotions, constructionist perspectives (e.g., Barrett and Russel, 2015) argue that emotions are dynamically constructed in the moment, emerging from the integration of bodily sensations, contextual appraisals, and the brain’s implicit knowledge of past experiences. From this view, a smile or laugh is not merely an expression of inner states, but an active, meaning-making event shaped by relational context.

Together, these insights contribute to underscore the complexity of nonverbal behavior in clinical settings. Additionally, they have pedagogical implications, highlighting that the learning of nonverbal relational competence demands experience reaching far beyond theoretical knowledge. While clinical supervision is widely recognized as essential for developing therapeutic skills, we know considerably less about how supervision can be structured to facilitate the acquisition of embodied, nonverbal competencies. Hence, we need empirical studies that explore how such nonverbal abilities emerge, are discussed, and shaped within supervision processes (Hill and Knox, 2013; Knox and Hill, 2021). In line with this, Hill et al. (2025) call for research that illuminates how both clients and therapists experience nonverbal phenomena in therapy, including crying, silence, and laughter. By focusing specifically on two of the most emotionally and socially complex nonverbal expressions, smiles and laughter, this study allows an in-depth analysis of these significant phenomena in psychotherapy. Of relevance not only to psychoanalysis but to all psychotherapeutic traditions, we ask: What can be learned from an empirical training study about the role of smiles and laughter in psychotherapy? And how might such insights inform the cultivation of nonverbal relational competence in psychotherapy education and supervision?

### Aims and research questions

In this study, we aim to illuminate the lived experiences of clients, therapists, and supervisors regarding how smiles and laughter were expressed, perceived, and understood throughout the psychotherapy processes. By foregrounding these subjective perspectives, we seek to deepen our understanding of the nuanced, interpersonal functions of smiles and laughter in clinical practice. Our aim is not to decode smiles and laughter as fixed emotional signals, but to understand how the clients and the therapists participate in the co-construction of meaning, affects, and relational dynamics, in line with Barrett and Russel’s (2015) argumentation. In doing so, we have undertaken to present a nuanced account of the embodied and relational textures of these phenomena in psychotherapeutic work. The object of the study is to answer three research questions: How did the dynamics of smiles and laughter unfold during the psychotherapy training processes? How did the therapists respond to clients’ expressions of smiles and laughter? How were smiles and laughter worked with in supervision?

## Materials and methods

### Design and study setting

This study represents an extension of a previous qualitative investigation exploring psychology students’ nonverbal relational skills in psychotherapy (present authors, submitted study). During the analytic work on the initial project, the first author gradually became aware of the distinctive and recurring presence of smiles and laughter in the data. These nonverbal expressions appeared to carry nuanced relational and affective significance that warranted further, dedicated exploration. The emerging focus led to the design of this study, which used the same data material as the previous study but aimed to specifically examine smiles and laughter systematically within the therapeutic interactions and the corresponding supervision sessions.

The study employed a naturalistic, multimodal qualitative design, combining two complementary approaches within each case: (1) micro-level observation of video recorded therapy and supervision sessions (e.g., Hoff et al 2024a), and (2) Interpersonal Process Recall (IPR) interviews (Elliot, 1986) with therapists, clients, and supervisors. Observations of the therapy sessions provided a detailed view of verbal dialogue and nonverbal exchanges within the therapeutic dyads, with an in-depth focus on smiles and laughter. Observation of the supervision sessions enabled an exploration of how smiles and laughter were addressed in reflective supervision dialogue.

From the video recorded material, the first author selected relevant video excerpts to guide the subsequent Interpersonal Process Recall (IPR) interviews (Elliot, 1986; see procedure for selection criteria). Transcripts from both the supervision sessions and IPR interviews were subjected to reflexive thematic analysis. The triangulation of participants’ reflections with observational data aimed to enhance interpretive robustness (Archibald, 2016; Levitt et al., 2017; Creswell and Poth, 2017). Five cases were selected to allow for exploration across therapeutic modalities and interpersonal contexts (Levitt et al., 2021; McLeod, 2010).

This study is part of a larger longitudinal research project called The Nordic Psychotherapy Study (NORTRAS), conducted at the Internal Clinic, Department of Psychology, University of (Oslo). The last year of a six-year profession-oriented degree program in psychology, student therapists provide individual psychotherapy under weekly three-hour group supervision. These students’ qualifications upon completion are comparable to doctoral-level training in clinical psychology (e.g., PsyD or PhD).

### Participants

The therapist group included four females and one male. All had prior experience with brief therapies, but none had engaged in more intensive psychotherapeutic formats. The supervisors (four women and one man) were all seasoned clinicians and supervisors, and all clients in the five cases were women. All participants were of Caucasian descent.

### Case selection and data material

To ensure diversity in therapeutic orientation, five cases were selected from the larger research project database: two metacognitive, two psychodynamic, and one integrative. Selection was informed by a prior study (present authors, submitted study) involving the same cases. In the original study, we chose these cases based on the richness of the video recorded material from both the therapy and the supervision.

- Case 1 (metacognitive): 13 therapy sessions (1 missing), 10 supervision sessions
- Case 2 (metacognitive): 13 therapy sessions (1 missing), 5 supervision session
- Case 3 (integrative): 12 therapy sessions (1 missing), 8 supervision session
- Case 4 (psychodynamic): 49 therapy sessions (4 missing), 18 supervision sessions
- Case 5 (psychodynamic): 45 therapy sessions (3 missing), 5 supervision sessions

The missing therapy sessions were, for instance, caused by technical issues and were distributed evenly throughout the duration of the training process. Since supervision was conducted in a group format, it was not feasible to precisely track the exact number if missing supervision sessions. However, the overall distribution of missed sessions appeared to be consistent over time, with no significant clustering or gaps at specific periods.

### The interviews

IPR interviews were conducted using selected video excerpts to evoke participant reflections on relevant therapeutic moments (Elliot, 1986; Meekums et al., 2016). For each interview, the first author prepared 4–6 therapy and 2–4 supervision excerpts. Clients only viewed therapy segments. The interviews followed a semi-structured guide (see Appendix), beginning with open-ended questions before gradually directing attention to the nonverbal dimensions of the selected material. As the interviews were originally prepared for a study of nonverbal relational competence in general, the interview guides did not include questions about smiles and laughter. However, when observing the video recorded material, the interviewer became aware of and interested in how smiles and laughter were expressed in the therapy sessions, and in the interviews later, she asked questions about this when considered relevant. When participants did not spontaneously reflect on nonverbal aspects, the interviewer employed gentle, reflective probing. These interventions were informed by clinical experience and aimed at fostering awareness rather than steering interpretation. The goal was to support participants in discovering their own meaning-making processes, while maintaining their agency and sense of ownership over the narrative.

For instance, in one interview, a therapist had not commented on her own non-response to a client’s laughter in a session excerpt. Rather than directly highlighting the omission, the interviewer asked, “What do you notice about how you responded there?,” leaving space for the therapist’s own observation. When this did not elicit further elaboration, a follow-up question was offered: “I noticed that the client laughed. Was there anything going through your mind in that moment?” This opened for a reflective dialogue about the therapist’s internal state, her decision not to mirror the laughter, and her intent to stay grounded in the client’s underlying emotional experience. Such interactions illustrate how the interviewer sought to gently attune to moments of potential clinical significance while trying to avoid imposing interpretative frameworks.

All interviews were video recorded, except for one therapist interview due to technical failure; to compensate, the interviewer immediately afterwards wrote a note documenting the content, which the participant later reviewed and approved. One client declined to participate in the interview phase but remained part of the overall study.

### Data analysis

Data were analyzed using reflexive thematic analysis (RTA; Braun and Clarke, 2006, 2013, 2019, 2023), emphasizing inductive, iterative engagement with the material. We drew on Finlay’s (2021) creative and embodied approach to reflexive thematic analysis, which we found particularly well-suited to exploring nonverbal phenomena. In line with Finlay’s perspective, we allowed our own embodied responses – moments of resonance, discomfort, or recognition – to inform how we constructed meaning. The analytic process proceeded through the following stages:

1. Without knowledge of their content beforehand, nine therapy sessions per case were randomly selected and reviewed (to cover different phases, three in early, middle, and late phases), along with all available supervision sessions. When observing the video recorded data material, the authors gradually became interested in how smiles and laugher came to expressions with a range of different qualities, both within each case and across cases. It piqued our curiosity regarding the various underlying emotions and relational dynamics that appeared embedded within these expressions. In some of the cases, there seemed to be a repetitive pattern in which the way smiles and laughter were expressed during the therapy process. In one case, for example, the therapist tended to laugh during sessions in a way that made us wonder whether she was nervous or felt insecure. In another case, the client tended to smile to her therapist in a way we experienced as ambiguous. Was it flirtatious, friendly, or simply an expression of how she was pleased to see him? Our multimodal method, combining our observations with IPR interviews, made it possible to add the informants’ own perspectives and experiences of these expressions.
2. All selected video content was transcribed using Whisper. Excerpts reflecting meaningful dynamics that were considered particularly relevant for the research questions were identified for use in IPR interviews. For the therapy sessions this included passages where the client and/or the therapist smiled or laughed in a way that made us curious about the underlying dynamics. For the supervision sessions the selected excerpts included passages where the supervision group discussed smiles and/or laughter. The selected passages were reviewed and discussed between both authors prior to interviews.
3. The first author conducted the interviews, case-by-case. When the first two interviews were finished, the second author read the transcripts, and the two authors discussed the interview technique and use of probing. She then conducted the rest of the interviews.
4. Following the interviews, rich and relevant transcript sections were selected for deeper analysis. This included passages where the informants gave detailed descriptions of their experience of nonverbal phenomena, including smiles and laughter.
5. The first author coded the material in NVivo, working case by case and developing interpretively rich codes. For each case, she began by coding the supervision sessions, followed by the three corresponding interviews. This sequential, within-case approach allowed for a deepened understanding of the dynamics across data sources (for examples illustrating the coding process, see Table 1). The second author coded two interviews and one supervision session, and the two authors compared and adjusted the further coding process.
6. Codes were grouped into initial theme candidates. Recurring patterns across cases informed theme refinement. The two authors discussed and modified the themes.
7. Final themes were defined and illustrated with selected excerpts (see Table 2).
8. First author completed a draft of the structure, which was subsequently approved by the second author. Afterwards, the first author wrote the final manuscript draft, incorporating refinements based on the second author’s contributions.

**Table 1:** Examples illustrating the coding process.

Quote	Code	Reflections	Theme
When I laugh it is as if I am devaluating myself. (Client)	When I laugh it is as if I am devaluating myself.	In the interview, the client seems to get in touch with how her laughter may cover some underlying feelings.	Smiles and laughter sometimes served to strengthen the therapeutic alliance, while at other times they functioned as emotion-regulating strategies or carried profound personal significance
T: *So there’s nothing that’s silly to say here. But it might still feel a bit scary* (smiles). C: *Yes* (laughts). T: *Yeah* (smiles slightly more broadly).	T: *So there’s nothing that’s silly to say here. But it might still feel a bit scary* (smiles). C: *Yes* (laughs). T: *Yeah* (smiles slightly more broadly).	The therapist demonstrates a subtle yet attuned responsiveness to the client’s emotional state. By smiling gently, she offers a nonverbal cue of warmth and reassurance without dismissing the client’s potential vulnerability.	The therapists intuitively tended to downregulate their responses to clients’ expressions of laughter, to modulate and contain the clients’ underlying emotions
It’s almost ironic how deeply she (the client) desired structure, while for me, offering it felt nearly impossible. (Therapist)	It’s almost ironic how deeply she (the client) desired structure, while for me, offering it felt nearly impossible.	The therapist seems to acknowledge how the dynamics in the therapeutic relationship may have affected her.	The way therapists handled laughter and smiles in the therapeutic setting seemed to be related to their degree of security and the quality of the therapeutic relationship
What was that smile to you? (Supervisor)	What was that smile to you?	The supervisor directly addresses the underlying meaning of the smile.	In supervision, smiles and laughter were not explicitly addressed as a distinct theme but occasionally surfaced spontaneously during sessions.

**Table 2:** Overview of the themes with quotes.

Themes	Quotes	Quotes	Quotes
Smiles and laughter sometimes served to strengthen the therapeutic alliance, while at other times they functioned as emotion-regulating strategies or carried profound personal significance	I remember we smiled quit a lot to each other. There was a friendly tone between us. (therapist)	When I laugh it is as if I am devaluating myself. (Client)	It does not have to be a problem with humor, like smiling or…but here (I think) we problematized it because it covered up something else. (Supervisor)
The therapists intuitively tended to downregulate their responses to clients’ expressions of laughter, to modulate and contain the clients’ underlying emotions	T: *So there’s nothing that’s silly to say here. But it might still feel a bit scary* (smil). C: *Y*. T: *Yeah* (smiles slightly more broadly).	C: *I told my father once, about the rape*. It actually made him embarrassed (laugs). T: (Gazes directly at the client with a serious, tender facial expression). *So your father felt embarrassed.*	C: *Yeah, it’s exhausting to feel so unwell, so I’m hoping that I can… talk myself into calmness.* T: *Yeah* (voice intonation rises). C: *So I’m kind of hoping for some* (laughs a little) *tricks* (laughs more loudly). T: *Yeah* (nods slightly, maintains eye contact with a serious but open facial expression).
The way therapists handled laughter and smiles in the therapeutic setting seemed to be related to their degree of security and the quality of the therapeutic relationship	She seemed secure. How she sat down in her chair, poured herself some water, as if she was saying here I am. I think it made me feel more secure. Here you are, and I welcome you. (Therapist)	I feel that I’m fairly secure in myself as a person (…). Not that I’m always like that. I do get nervous too. And when I watch myself here, I can see that at times I try to downplay things a bit. (Therapist)	It’s almost ironic how deeply she (the client) desired structure, while for me, offering it felt nearly impossible. (Therapist)
In supervision, smiles and laughter were not explicitly addressed as a distinct theme but occasionally surfaced spontaneously during sessions.	Several times, I have said to students: “Could it be that you do not need to be so cheerful all the time? Perhaps what is more important is just being present.” (Supervisor)	What was that smile to you? (Supervisor)	She cried, but then she started laughing. (Group member)

### Research team and reflexivity

The team consisted of two Caucasian middle-aged female clinical psychologists and psychoanalysts. The first author, also a former choreographer and dancer, brought a heightened sensitivity to embodied expression. Both researchers’ psychoanalytic training influenced their attention to underlying emotional and relational processes. In line with a reflexive thematic analysis approach (Braun and Clarke, 2006, 2013, 2019, 2023; Finlay, 2021), we engaged actively and explicitly with our own subjectivities throughout the analytic process. Our professional backgrounds offered valuable resources for noticing and making sense of subtle, embodied, and relational aspects of the material. These perspectives allowed us to attune closely to nonverbal expressions, movement, and affective nuances.

Concurrently, we recognized that our common psychoanalytic orientation might influence, and at times, limit our interpretations. A central example of this reflexive work was our repeated discussions around what we came to describe as an “uncertain smile.” We asked ourselves: What leads us to perceive a smile as uncertain? Is it related to the patterns of facial muscle tension, a lack of alignment with verbal content, or something else entirely? These reflections prompted us to critically examine how our own clinical training might influence our perception and meaning making. To mitigate the risk of theoretical narrowing, we engaged in ongoing dialogue with a colleague from a different theoretical background. These conversations served as a productive counterpoint, challenging our assumptions and enriching our analytical process. In this way, reflexivity became both a methodological commitment and a dynamic practice of negotiating between our professional expertise and a genuine openness to alternative perspectives.

### Ethical considerations

The study was approved by the university’s Data Controller and received exemption from the Regional Ethics Committee. All participants gave informed, written consent and had opportunities to review and comment on the material. No dual relationships were present in the analyzed cases.

## Results

The RTA analysis of the supervision sessions and the IPR interviews yielded four themes: 1. Smiles and laughter sometimes served to strengthen the therapeutic alliance, while at other times they functioned as emotion-regulating strategies or carried profound personal significance; 2. The therapists intuitively tended to downregulate their responses to clients’ expressions of laughter, to modulate and contain the clients’ underlying emotions; 3. The way therapists handled laughter and smiles in the therapeutic setting seemed to be related to their degree of security and the quality of the therapeutic relationship; and 4. In supervision, smiles and laughter were not explicitly addressed as a distinct theme but occasionally surfaced spontaneously during sessions.

Due to the sensitivity of the data, we have masked the specific cases from which the clinical material is drawn to protect the anonymity of the informants. Furthermore, to preserve the complexity of the material and to highlight the triangulation of the three informants’ perspectives in each case, we have chosen to focus on fewer, in-depth examples rather than several shorter ones illustrating each theme. In what follows, we describe each theme and provide examples to illustrate them.

### Theme 1. Smiles and laughter sometimes served to strengthen the therapeutic alliance, while at other times they functioned as emotion-regulating strategies or carried profound personal significance

When observing the video recorded therapy sessions, the authors were drawn to how smiles and laughter came to expressions in qualitatively different manners in different situations. Not unexpectedly, these affective expressions seemed to follow some regular patterns in each case. In one case, for example, the therapist and the client would smile at each other in a particularly friendly manner. These smiles appeared to express friendliness, empathy, openness, and a mutual interest. Moreover, we noticed that these smiles seemed to be congruent with the verbal content of the dialogue. The therapist in this case, in the beginning of her interview, spontaneously said *I remember we smiled quit a lot to each other. There was a friendly tone between us.*

The following example is from another case. In one session, the client and the therapist are discussing the client’s relationship to conflict. Here, the client recounts an argument with her partner:

C: It was… my partner and I had an argument. And then I lay down next to him and put my arms around him. He then said, “Could you move back a little?” and I thought he meant that I should move away from him again (laughs a little, with smiling eyes). But I had misunderstood, he just meant could I shift my position (laughs louder, with smiling eyes).

T: Yes (laughs, with smiling eyes).

C: And I got really annoyed (laughs, with smiling eyes).

T: Yeah (laughs, with smiling eyes).

C: And then we talked about it for a long time, down to the tiniest detail.

T: Yeah (nods, looks directly at the client with a serious, empathetic facial expression).

In this brief exchange, the client and the therapist appear to be in strong emotional contact with each other. The client’s use of gentle laughter, suggests that she is emotionally present and at ease in sharing the patient’s experience, even when she is describing a challenging moment. The therapist responds with a matching emotional expression, laughing softly. This attuned mirroring clearly fosters a sense of mutual understanding and emotional resonance.

As the client moves from light humor into more detailed reflection, the therapist shifts her own nonverbal stance; she stops laughing, nods, and meets the client’s gaze with a serious, empathetic facial expression. This subtle transition indicates that the therapist is closely tracking the client’s emotional tone and content, adjusting her own emotional expression accordingly. Such responsiveness reflects a high degree of attunement, as the therapist fluidly shifts between resonating with the client’s light tone and creating a supportive space for deeper exploration. The moment illustrates how ongoing, modulated affective synchronization can support a sense of being seen, understood, and emotionally contained in the therapeutic encounter.

However, in the data material, we found several examples were smiles and laughter came to an expression with a different quality and seemed to us to regulate some underlying uncomfortable emotions, in the client, the therapist, or between them. One of the clients, for example, sometimes started to laugh during sessions, without something funny being said. In the interview with this client, she expressed that she in retrospect believe that during the therapy her laughter covered some difficult emotions: *There were some difficult feelings underneath, but I was not aware of them. Things are not difficult when you laugh.*

In another case, the following scene took place:

C: In gym class at school, I could faint easily, and the others thought it was funny (starts crying) now I just… I start crying so easily (starts laughing).

T: Yes. That’s completely okay! (lips curve slightly upward in the hint of a smile).

C: (Laughs) Yeah (smiles at the therapist).

In the interview, when we had watched this passage, the client commented on her laugher*: I do not think I was aware of it then, but when I see this now, I think I was nervous. When I laugh it is as if I am devaluating myself.* The therapist, in her interview, when we had watched the same excerpt, said *I notice how she cries, but then she laughs as well. It is like she is defending herself. She starts laughing, but she is really scared.* In her further associations, she reflected on her own way of being with the client: *I think I was a bit overwhelmed. I can see that I almost try to ease the atmosphere. I noticed that I for a second almost smiled a bit.* Here, the therapist demonstrates that she retrospectively recognized a tension in the therapy setting. She used her observation of her own tendency to smile to reflect on her own ability to tolerate the client’s emotional pain. Importantly, the therapist’s facial expression did not develop into a full smile in the actual situation. This may indicate that she was aware of her own emotional responses and was able to modulate them to better attune herself to her client’s emotional pain.

In another case, at the beginning of the first session, the client and the therapist enter the therapy room together. The therapist is holding some papers in her hand, and as she sits down, she places the papers on her lap. The client is carrying a bag, and as she sits down, she places the bag next to her chair, and bends over towards her bag, apparently looking for something. Now, the following dialogue takes place:

T: Hi there! (Looking down at her papers, starting to laugh at the end of the sentence).

C: (Still bending over and facing her bag) Hi. (Sits back in the chair, meets the therapist’s gaze with a little laughter).

In the therapist interview, when we had seen this excerpt, the therapist immediately said *I notice that I have these papers on my lap. I think that was a bit rejective, in a way.* The client, after seeing the same scene, said:

I saw that she felt insecure about me, because I was not very present at the beginning. I was not sitting there waiting for her, I was doing my own things. It looks like she gets insecure, like when will I attune to her? She does not look at me, but I don't notice, because I am doing my own things (….) She did not offer much framing or containment of my emotional experience. But that may be because she does not understand how important that is for me. I am stressing around with everything, so…

The supervisor, having watched the same passage in his interview, said:

There is something about (the therapist’s) tolerance for negative affect that could be a bit challenging. Saying “Hi there”, contra sit down, calmly, set the agenda. It does not have to be a problem with humor, like smiling or…but here (I think) we problematized it because it covered up something else. (The client) is doing her own things with her cell phone. And (the therapist) is beginning the session saying “Hi there” without waiting for the contact to be established between them. She has not really started the session. What she could have done, was to wait for the client to put her phone away and then start the session. So, this is not the best beginning of a session. The premises are not set for the work they is supposed to do. To get an eye contact first or at least try to get an eye contact. But here one gets curios about what is going on between them.

In the same case, therapy session 4, there is a scene again in the very beginning of the session, where the therapist asks the client whether it is ok that they use this session to take a closer look at a (metacognitive) model. The client says yes and tells the therapist that she is more comfortable when the sessions have a clear structure, laughing while she talks. In the following scene, her facial expression is tense and anxious, and she gazes out at the room, glancing in the direction of the therapist, without meeting her gaze. At one point, she is pulling the arms of her sweater, looking uncomfortable. The therapist sits calmly in her chair. She is folding her hands, and gazes towards the client:

C: I can feel stressed and take a lot of responsibility to like… to come up with something that satisfies her (nonverbal signs indicate that she refers to the therapist).

T: Mm.

C: Yes, I can stress with that…that I don’t have enough to come up with.

T: But do you think you would be able to tell me, if you felt it like that here? (appears not to pick-up that the client had referred to her)

C: Yes… yes, yes (laughs). Sure, yes, sure (withdraws her gaze, leans back in her chair, away from the therapist).

T: (Laughs) Yes.

The client, after seeing this passage in her interview, said *Well, it seems to me that I do not feel free to do it (…) Here she could have said: “Are you not sure about it?” She could have explored it more. She laughs a little and I think she understands that I feel insecure about it.* The therapist, after seeing the same scene, said:

T: She says yes, but at the same time she says no.

I: What do you think of your way of responding to her?

T: I am insecure whether I reached her.

I: Do you believe you accurately captured the nuance of her simultaneous affirmation and negation in that moment?

T: I think so. I don’t know if she felt more secure, I don’t think so.

In the supervisor interview, we watched the same passage, and the supervisor said:

When they started talking about the case formulation, you can see that the client is withdrawing. She pulls her sweater, and she is obviously nervous. So, she gives some signals related to the therapy, and (the therapist) captures this, and asks a very important question. The client is avoidant in her response, and then (the therapist) responds with laugher. It is not something to laugh about, and it is interesting that it happens. I don’t know how this developed further, but it would be best not to laugh and rather say: “I saw you were laughing when you said that, and I am not sure whether you meant yes”. This would be nice to comment on, but instead I think (the therapist) became nervous.

In another case, we noted that the therapist and the client often smiled to each other in a particularly warm way, especially at the beginning and ending of sessions. We were intrigued by the client’s smile, which came across as both flirtatious and inviting, yet at the same time somewhat shy and reserved. The therapist in this cased expressed in his interview that he felt the client smiled to him in a way that made him want to see her again.

Another repetitive pattern in this process was that the client ended each session by saying *See you on Friday*, while smiling in her idiosyncratic way. In every session, the therapist confirmed this by saying *Yes, we do,* or repeat *Yes, see you on Friday.* At one point during the therapy, they talked about this scenario, leading the client to reveal that she as little had a terrifying phantasy that her mother would kill her as she was asleep. She developed a strategy where she used to whisper *See you tomorrow* as her mother left her bedroom in the evening, believing that this would hinder her mother from taking her life.

In the interview with the client, the interviewer asked her about her way of ending the sessions and commented that it almost seemed like a ritual. The client was very moved by this, and seemed to discover, then and there, that she during the therapy had been afraid not to see her therapist again, in the same way as she had been terrified not to see her mother again as a child. Hence, one could ask whether her smile expressed several underlying meanings. She smiled in a way that made the therapist want to see her again. Underneath lied her fear of not being able to see her therapist again, which again reflected her child anxiety of not being able to see her mother again.

In sum, this theme underscores the complex role of smiles and laughter in psychotherapy. On one hand, they appeared to foster warmth, safety, and relational attunement—hallmarks of a strong therapeutic alliance. Simultaneously, they functioned as subtle regulators of emotional intensity, both for clients and therapists.

### Theme 2. The therapists intuitively tended to downregulate their responses to clients’ expressions of laughter, to modulate and contain the clients’ underlying emotions

During the analysis, we were struck by how contagious smiles and laughter appeared to be within the therapeutic encounters. When one participant smiled or laughed, the other would almost invariably follow. However, we observed that smiles and laughter were most often initiated by the clients. When therapists responded with laughter, their expressions tended to be softer, more restrained, and less intense than those of the clients. This pattern gave us the impression that the therapists were actively modulating their own emotional expressions, as well as the clients’ expressions. Rather than fully matching the client’s affective display, they seemed to calibrate their responses in a way that maintained emotional connection while also preserving a sense of therapeutic containment.

In what follows, we will give a brief example from the beginning of a first session. The therapist and the client are discussing the therapy and how it will unfold:

T: So, there’s nothing that’s silly to say here. But it might still feel a bit scary (smiles).

C: Yes (laughs).

T: Yeah (smiles slightly more broadly).

In this exchange, the therapist demonstrates a subtle yet attuned responsiveness to the client’s emotional state. By smiling gently while acknowledging that speaking in therapy might feel “a bit scary,” she offers a nonverbal cue of warmth and reassurance without dismissing the client’s potential vulnerability. When the client responds with a laugh which seemed a bit insecure or uncomfortable, the therapist appears to register this nuance. Rather than mirroring the client’s laughter, she maintains a calm presence, smiling slightly more broadly but refraining from laughing herself. This modulation of her own nonverbal expression can be seen as an effort to contain the emotional tone of the moment and to remain anchored in the underlying affective meaning, rather than being drawn into a potentially defensive or disarming display. In doing so, the therapist provides a steadying and validating presence, signaling both acceptance and emotional containment, key elements in fostering a safe therapeutic space.

In examples like this, we got the impression that the therapists automatically modulated the quality of their laughter, and we questioned whether they sensed the clients’ underlying emotions in the same way as we understood them. Furthermore, we were curious about the therapists’ awareness of their modulation. During the therapist interviews, each participant supported this idea in various ways, noting that they believe their responses are a learned behavior. One said *as a human being, I think it is very natural to respond by smiling when another person is smiling to you. So, I think it is a learned response.* Another said *I think it is learned.* We were also interested in whether the therapists thought they had learned about this during the psychology study program. When asked about this in the interviews, none of the therapists remembered a concrete course or a concrete situation where this was discussed. One of the therapists stated *I’m not sure. Sometime during the study program?* Another said *I do not remember exactly. But I have a feeling that we talked about it.*

Two of the therapists were distinctive in that they did not necessarily respond to the client’s laughter by laughing themselves. For example, in one therapy session, the following dialogue took place:

C: I told my father once, about the rape. It actually made him embarrassed (laughing).

T: (Gazes directly at the client with a serious, tender facial expression). So, your father felt embarrassed.

In the subsequent supervision session, the group watched this excerpt from the therapy. In the discussion afterwards, one of the group members commented the client’s laughter. The therapist immediately reacted, and asked the group *What did I do? I hope I did not start laughing as well.* Hence, in supervision, she did not remember this moment but was highly conscious of her own response, and afraid that she had met her client’s laughter with laughing herself. No one in the group had noticed the therapist’s reaction, but upon rewatching the excerpt, they concluded that she did not laugh in her response.

During our observations of this therapy session, we were particularly interested in whether the therapist’s response was a conscious decision. In the interview with the therapist, she was asked about this, and replied *Well, it was not funny. I am glad I did not laugh; I believe that would have been inappropriate. I am not sure if I was fully aware of it at the time (…) I think I was.*

In the second case, where the therapist often held back laughter when the client laughed, the following dialogue took place, in session 1:

C: Yeah, it’s exhausting to feel so unwell, so I’m hoping that I can… talk myself into calmness.

T: Yeah (voice intonation rises).

C: So I’m kind of hoping for some (laughs a little) tricks (laughs more loudly).

T: Yeah (nods slightly, maintains eye contact with a serious but open facial expression).

In the interview with the client, when we had watched this excerpt, she said:

I feel that she takes me very seriously (…) whether I was thinking about that in the moment, I’m not sure. But one of my biggest fears is being laughed at. So, the fact that she is so steady and affirming… I mean, it’s not funny. It’s more a reaction on my part.

Hence, the client clearly experienced the therapist’s choice not to mirror her laughter as a sign of being genuinely taken seriously. In the interview with the therapist, we had watched the same excerpt, and the following dialogue took place:

T: What I notice is that she is a bit restless, and that she laughs. She is actually talking about something that is really important to her. And then she laughs.

I: I notice that when she laughs, you do not laugh.

T: Maybe I do not. I guess that is a good thing. It shows that there is nothing funny about it. I take what she is saying seriously.

The therapist shows an intuitive capacity to attune to the client’s underlying emotional state, choosing to respond to that level rather than simply mirroring the superficial affective display.

In sum, this theme emphasizes that the therapists typically downregulated their own laughter in situations where laughter seemed to conceal uncomfortable feelings. The analysis conveys that these responses were carried out intuitively, rather than as the result of a rational decision-making process. At the same time, the therapists expressed in their interviews that they think they were aware of what they were doing in the moment. Even though these responses seemed to occur automatically or intuitively, several therapists stated that they believed they had learned this during their psychology training. However, they were unable to pinpoint exactly when or where.

### Theme 3. The way therapists handled laughter and smiles in the therapeutic setting seemed to be related to their degree of security and the quality of the therapeutic relationship

The data analysis conveyed a pattern in the material: The therapists who seemed more secure in the therapy setting also seemed to be more in control of how they responded to the clients’ laughter. The therapists who felt more insecure, on the other hand, tended to respond to the clients’ defensive or emotion-regulating laughter with a synchronized laughter, characterized by less modulation. Equally important, the therapists’ insecurity seemed very much to be related to the dynamics in the therapeutic relationship. Therefore, feelings of insecurity do not solely indicate something intrinsic to the therapist; rather, they may primarily reflect challenging or uncomfortable emotions arising from the therapeutic relationship itself.

In theme 2, we described that two of the therapists avoided to laugh in response to their client’s laughter, which to the therapists appeared as the client’s defensive and/or regulating reactions. In the very beginning of the interview with the therapist in the case first described, when she was asked about what comes first to her mind thinking back at the therapy, she said *It is her. I remember her very well. Her way of being, how she entered the room, how we were in the room together*. Later in her interview, after the topic of the study was introduced and we discussed the nonverbal interaction between herself and the client, she said *she seemed secure. How she sat down in her chair, poured herself some water, as if she was saying here I am. I think it made me feel more secure. Here you are, and I welcome you.* The therapist further told the interviewer that she worked deliberately with herself to increase her security both as a person and as a therapist during the psychology study program. She participated in a group of students that met regularly to talk about themselves and challenges they faced in their role as therapists.

In supervision, this therapist seemed particularly able to come forward with her own vulnerability. In the second supervision session, after the group had watched the second therapy session on video, she asked the group *Am I too calm, too careful?* Here, she demonstrates how she dared to expose her vulnerability. In her interview, when she reflected on her own feelings before the therapy process started, she said *I remember thinking that she (the client) seemed to be so talkative and lively. And I though, oh, do I have to be like that as well? Or can I be more myself, a bit calmer, which is my natural way of being. And I said to myself, hopefully that (being myself) can be something in itself.* Hence, this therapist appeared very intuitive in her thinking about how she could attune to the client in her own way on a bodily level. Furthermore, she gave the impression of being highly aware of how her own bodily presence and way of being might affect the client.

In the other case where the therapist also withheld to respond to the client’s laugher by laughing herself, the therapist expressed in her interview that she felt relatively secure as a person:

I feel that I’m fairly secure in myself as a person (…). Not that I’m always like that. I do get nervous too. And when I watch myself here, I can see that at times I try to downplay things a bit. But… I’m not particularly afraid of people feeling a lot, or of them crying. I also remember thinking that it could feel a bit overwhelming… I can almost feel it now as we watch (the excerpt) that it was intense. But at the same time, I don’t think I experienced it as dangerous. And as a psychologist, I think… I do think it’s our job to tolerate (the feelings of) the patient.

The therapist reflects on having a relatively stable sense of self, acknowledging both confidence and vulnerability. She recognizes moments where she attempts to downplay the emotional intensity of the situation yet also expresses a clear stance; she is not afraid of strong emotions or of clients crying. Although she recalls the experience as intense, she did not perceive it as threatening. Importantly, she articulates a professional ethic, that as psychologists, it is the task to tolerate and contain the client’s emotional expressions.

This stance may help explain her notable capacity to modulate her own nonverbal responses during the session. Rather than automatically synchronizing to the client’s laughter, she appears to remain attuned to the underlying emotional content and to respond in a grounded, containing manner. Her ability to hold emotional space without becoming emotionally overinvolved or defensive seems to reflect both personal stability and a well-developed sense of therapeutic presence. This, in turn, allows her to meet the client with seriousness and emotional availability, even when the affective expressions on the surface are ambiguous or potentially disarming.

In the case described in theme 1, where the therapist tended to respond in a synchronized way to the client’s emotion-regulating laughter, the therapeutic relationship seemed to be characterized by more tension and insecurity. In the beginning of the interview with the client in this case, she expressed that she liked her therapist a lot and felt that she experienced the therapeutic relationship as positive. She said *I thought she was really sweet, and she gave me this good feeling that she was very interested in helping me.*

However, when she continued, she also expressed that she sensed an insecurity in the therapist: *I think I sometimes noticed that she seemed a bit insecure (…), maybe that she was not completely sure about the method.* When we had watched an excerpt from therapy session 2, the client further questioned whether the therapist’s insecurity could be related to her own way of being in the setting: *What I see now, is that maybe she gets a bit insecure about me. Because I see that I am not really present. I did not sit there waiting for her, I am more into my own things in a way.* After watching a later excerpt from the same session, she remarks *I feel that I appeared emotionally unaffected as I spoke. But that’s likely because I’ve talked about the same things so many times (in other situations).*

Throughout our observations of the therapy sessions in this case, we perceived that the therapist was deliberately endeavoring to sustain her composure, even as the client spoke extensively, at a rapid pace, and with a rather frantic style. In the beginning of her interview, the therapist expressed:

I remember noticing her stress. She talked a lot, and very fast (….) I recall a fluctuation between what am I saying, what am I expressing nonverbally, and what am I doing (…) I remember how important it felt to radiate a sense of calmness and safety. It was something she clearly needed.

Despite the client’s restless and fast-paced communication style, the therapist appeared able to maintain a steady and composed presence throughout the sessions. In her interview, she further expressed how she during the therapy sessions was consciously working to uphold a sense of calmness, even in the face of a somewhat frenzied interpersonal rhythm. She further recalls being acutely aware of the client’s stress and her own efforts to manage both her verbal and nonverbal responses. Her statement above suggests a high degree of attunement to the client’s emotional needs. Rather than becoming swept up in the client’s tempo or affective urgency, the therapist reflects on an internal monitoring process, shifting between awareness of what she was saying, what she was expressing nonverbally, and what she was doing. This self-reflective stance indicates an active regulation of her therapeutic presence, aimed at offering a stabilizing counterbalance to the client’s rapidity.

However, in the beginning of her interview, the therapist also expressed that she felt it was challenging to handle the client’s hectic style: *I remember feeling that she was difficult to contain, at least in the beginning (…) There were a lot of things in my head simultaneously. It made it demanding to be present and focus on the body.* As the conversation progressed, she articulated an insightful paradox: *It’s almost ironic how deeply she (the client) desired structure, while for me, offering it felt nearly impossible.*

Later in the interview, when the therapist had seen an excerpt from therapy session 2, she immediately said *I think I am rather tense. I can sense it in my voice and in my breath. I do not feel very comfortable in that chair yet. Probably, I am quite nervous.* Moreover, in line with the client, she expressed that she remembers thinking that the client was not very much in contact with her own emotions: *She did not relate very much to her own feelings. She was telling stories that she had told a lot of times before. It was difficult to help her turning her focus inwards. I thought: “How can I help her with that?”*

Later in her interview, this therapist further expressed that she remembers feeling that it became challenging for her when the client suddenly appeared more in contact with her feelings:

I remember thinking that this is a turning point, when she once started crying. This is good (…) But I also remember it as a bit difficult. We discussed that in supervision, what is the right thing to do when someone gets in contact like that with difficult feelings. I still find it a bit difficult. In a way, it is what you are sitting there all the time waiting for, and then, when it finally happens, it is not so easy to know what to do.

The therapist here offers a nuanced account of the challenges involved, particularly for a novice practitioner, in navigating clients’ shifting modes of expression, especially when oscillating between emotional distance and intense affective breakthroughs. To what extent the tension in the therapeutic relationship stemmed primarily from the therapist, the client, or the dynamic emerging between them is, of course, difficult to determine with precision. What we found both important and compelling was that *both* the client and the therapist expressed a sense of uncertainty in the room. At the same time, it appeared to us that the therapist occasionally struggled to modulate her own expressions of smiling and laughter.

In sum, this theme illustrates that, in the data material, we found a relationship between therapists’ degree of security and their responses to clients’ smiles and laughter. The therapists who seemed more secure on video, and in addition, in their interview expressed a certain level of security in the therapist role, seemed more able to modulate their own bodily and verbal responses towards their client. The therapists who seemed more insecure on video, and/or expressed an insecurity in the therapist role in their interview, seemed less able to consciously modulate their own responses, both verbally and nonverbally. This suggests that laughter and smiles are not only tools of regulation and connection, but also indicators of the underlying relational quality.

### Theme 4. In supervision, smiles and laughter were not explicitly addressed as a distinct theme but occasionally surfaced spontaneously during sessions

In the IPR interviews, all the supervisors expressed that they think work with the nonverbal is important. One said *I believe this is very important. As a supervisor, one of my guiding principles is to help students become aware of things they may not yet realize themselves.* One of the supervisors conveyed that she did not believe they worked explicitly in supervision with nonverbal cues, whereas four of them conveyed a belief that they had worked with the nonverbal level of the dialogue. At the same time, all the therapists expressed that they could not remember any concrete work with the nonverbal part of the dialogue in supervision.

Observations of the supervision sessions revealed no examples where the groups explicitly addressed therapists’ and/or clients’ expressions of smiles and laughter as a distinct topic. Neither did they discuss whether, how, or why the therapists modulated their own expressions of smiles and laughter. However, some examples were found were the supervisor and/or the supervision groups addressed the therapists’ nonverbal ways of being in the therapy setting, and where this spontaneously led to a discussion of smiles and laughter. In one case, the supervisor demonstrated, both in supervision and in her interview, how she deliberately worked with the therapist’s behavior in the therapy setting. In supervision, the group discussed how the client talked quickly with a high pitch in the tone of her voice. They further talked about how the client, when she got in touch with some difficult emotions, started crying, and how the crying had a tense quality and quickly could turn into a laugher. However, they did not discuss the potential emotions underlying these expressions.

In supervision session 3, after the group had watched therapy session 3 simultaneously on a monitor, the group discussed the session while the therapist was listening to their discussion. The supervisor said *It is fascinating how she (the therapist) manages to handle and accept the client’s stress and chaos and contain it in a very accepting way. It is very nice and therapeutic.* In her interview, after watching a passage from therapy session 1, the supervisor said:

She (the therapist) has a lively character (…), and tends to speak with a high pitch, like many young women. In this way, she resembles the client. Still, I believe (the therapist) noticed the client’s hectic intensity, (…) and (she) has a bit more ore in her voice, as I often say. She has a substance in her voice, which I think is good.

This supervisor was particularly explicit on how she tries to teach the students that they primarily need to be emotionally present in therapy, and how this involves helping the students to regulate their nonverbal expressions. In her interview, she said *Several times, I have said to students: “Could it be that you do not need to be so cheerful all the time? Perhaps what is more important is just being present”*.

At the end of her interview, this supervisor expressed that she remembered seeing the therapist together with her baby at one point at the time when the therapy process took place. She said *I remember noticing that she had this tender expression together with her baby, and I was thinking, I would want her to bring a bit more of that into the therapy room. I think I told her. I am not completely sure, but I hope I did.*

In other examples, the supervisors addressed the personal and the relational meaning behind clients’ smiles and laughter. For example, in the case described earlier where the client’s smile seemed to cover some deep personal experiences (see theme 1), the supervisor in the subsequent supervision explicitly addressed the client’s smile, saying *What did that smile mean to you?* In the further discussion, the group reflected on several possible ways to understand the client’s smile. One group member highlighted the smile as a possible expression of her attachment to the therapist. Another wondered whether the client was trying to avoid burdening the therapist with her own difficult emotions.

In sum, this theme highlights that in the IPR interviews, all the supervisors expressed that they think work with the nonverbal is important, and four of them also conveyed a belief that they had worked with the nonverbal, including smiles and laughter, in the supervision process. However, observations of the supervision sessions did not convey concrete work with expressions of smiles and laughter. Moreover, all the therapists expressed that they could not remember any concrete work in supervision with the nonverbal level of the therapeutic dialogue. In our data, some examples were found where therapists, supervisors, and group members spontaneously addressed in supervision the quality of smiles and laughter in therapy sessions. However, no examples were found where the supervision groups worked deliberately with modulation of therapists’ expressions of smiles and laughter.

## Discussion

The aim of this study was to investigate how smiles and laughter were expressed, modulated, experienced, and reflected upon in five psychotherapy training processes. Through reflexive thematic analysis of video recorded supervision sessions and interpersonal process recall (IPR) interviews with the clients, therapists, and supervisors in each case, four themes were developed that illuminate the affective and relational significance of smiles and laughter in psychotherapy and the pedagogical potential of addressing these specific nonverbal dynamics in training.

First, smiles and laughter at times served to primarily strengthen the therapeutic alliance, while in other instances they appeared as strategies for regulating affects or expressing personally significant material. Second, therapists often responded to clients’ laughter with a subtle downregulation of their own corresponding expressions, suggesting an intuitive effort to modulate and contain the clients’ emotional experience. Third, the ways in which therapists engaged with these nonverbal cues appeared closely linked to their sense of internal security and the quality of the therapeutic relationship. Finally, although smiles and laughter were not explicitly addressed in supervision to support the therapist’s ongoing work, attention to these nonverbal phenomena occasionally surfaced spontaneously in the discussions.

The title of this article, *The Unbearable Lightness of Laughing*, plays on Milan Kundera’s renowned novel *The Unbearable Lightness of Being* (Kundera, 1984/1999), evoking the existential tension between lightness and weight, between meaning and evasion (Kabir, 2010). In our study, this metaphor captures how laughter, while seemingly light and connecting, could simultaneously carry the weight of emotional avoidance. Hence, laughter sometimes could transform moments of potential emotional depth into something unbearably light—pleasant, acceptable, and yet fundamentally distancing. Thus, what appeared as a fleeting moment of levity could, in fact, serve as a defense against precisely what therapy is meant to bear witness to.

The therapists in our study demonstrated varying capacities to modulate their own expressions of smiles and laughter. At the same time, several of the clients indicated that there were situations during therapy where they did not feel emotionally contained when the therapists laughed. Moreover, one client clearly expressed that when the therapist responded to her laughter in a modulating manner, or refrained from laughing altogether, she felt genuinely taken seriously and emotionally acknowledged. Hence, our results seem to align with previous research findings, highlighting that therapist mimicry of client’s emotional expressions do not necessarily lead to positive outcomes (Benecke and Krause, 2005; Dreher et al., 2001). However, in our study, the therapist’s capacity to modulate their affective expressions appeared linked to their level of internal security or uncertainty in the clinical moment, pointing towards a need of supervision. At the same time, our results indicate that this need for supervision may not be adequately met.

Notably, while several therapists described the ability to modulate smiles and laughter as something they had learned, none could clearly recall when or how they acquired this skill. This suggests that such competencies may be implicitly absorbed rather than explicitly taught—potentially a form of procedural learning tied to broader processes of affect regulation. However, these results also raise the question of whether the therapists in our cases could benefit from more explicit attention to smiles and laughter in their training processes. Generally, supervisors may encourage trainees not only to notice when they laugh or smile, but to ask themselves: What was I feeling in that moment? What was I managing, modulating, or avoiding? This kind of reflective stance supports the development of what Lemma (2016) refers to as mentalized affectivity, the capacity to think and feel simultaneously, which allows for a responsive yet contained therapeutic presence. In doing so, therapists cultivate not only technical skill but also emotional depth and relational integrity.

Our findings align with psychoanalytic literature emphasizing the therapist’s embodied subjectivity in the clinical encounter. Freud (1923) famously stated that “the ego is first and foremost a body-ego,” underscoring its foundation in bodily sensations rather than abstract functions (p. 26). He further emphasized that “the ego is ultimately derived from bodily sensations,” pointing to the physical origins of our sense of self (p. 26, note). In line with this, Ogden (1994) argues that the therapist’s bodily experience is not outside the therapeutic field but is part of the intersubjective matrix through which meaning and emotion are co-created. This speaks to Winnicott’s (1960, 1965, 1971) foundational concept of the *holding environment*, where the therapist’s reliable emotional presence serves as a psychological equivalent to the mother’s physical and emotional holding of the infant. Holding, in this sense, is not limited to verbal reassurance; it is enacted through tone, posture, rhythm, and facial expression, forming a nonverbal scaffolding that allows the client to feel psychologically safe.

Similarly, but also slightly different, is Bion’s (1962) notion of *containing.* This concept refers to the therapist’s capacity to receive, mentally process, and transform the patient’s raw emotional expressions, often experienced as overwhelming or confusing, into something more tolerable and thinkable. Hence, whereas Winnicott’s (1960) concept of *holding* refers to the foundational safety necessary for psychological growth, containing involves a more active internal work on the therapist’s part; the emotional digestion of what the patient cannot yet bear alone. Containing thus represents a crucial function in affect regulation and mentalization, allowing unformulated experience to be symbolized within the therapeutic relationship.

Following Bion’s thinking, the therapist’s ability to remain in connection with the emotions the client tries to avoid, rather than reflexively mirroring every emotional expression, such as smiling or laughing in response, constitutes an act of containment. It allows the client’s affect to be symbolized and thought about. Hence, therapist non-synchrony, when modulated and intentional, can itself be therapeutic, functioning as a form of affective regulation that supports integration rather than escalation or dissociation.

In this respect, our study contributes to ongoing debates in the empirical research literature on nonverbal synchrony (Bar-Kalifa et al., 2023). Some suggest nonverbal synchrony is universally beneficial (Gregorini et al., 2025; Nyman-Salonen et al., 2021a; Ramseyer, 2020), while others find it can be neutral or even countertherapeutic depending on context (Atzil-Slonim et al., 2023; Jennissen et al., 2024; Nyman-Salonen et al., 2021b; Koole and Tschacher, 2016; Ramseyer and Tschacher, 2011). In our results, we saw that therapist’s synchrony, when the client’s laughter seemed to represent an avoidant strategy, sometimes had a contra-therapeutic effect. Rather than prompting the therapeutic bond, such synchrony seemed to reinforce avoidance. In contrast, when the therapists remained emotionally grounded and downregulated own laughter or smiles in a subtle, differentiated manner, this seemed to represent a more attuned form of synchrony that increased the client’s feeling of being understood.

A key strength of this study lies in its research design. By applying a complex, multimodal qualitative approach to a highly specific topic, it becomes possible to illuminate the layered nature of clinical dialogue. Psychotherapeutic processes unfold on two interrelated levels; the observable, external expressions that can be seen or heard, and the underlying emotional dynamics that must be inferred and interpreted. Our results indicate that this layered complexity was not always fully acknowledged or utilized in the supervision discourse. Smiles and laughter were rarely addressed explicitly, and when they were, the discussions often reflected a limited awareness of this dual nature.

In some cases, the supervision groups remained primarily on the level of observable behavior, with little exploration of the underlying affective processes. In others, they moved too quickly to interpretation, bypassing the careful observation that might have grounded a more accurate or meaningful understanding. It is imperative to exercise caution when generalizing based on a limited sample. Nevertheless, a pattern emerged across cases. The tendency to move too quickly to interpretation was more pronounced in the dynamic therapy groups, whereas in the integrative therapy groups, moments of nonverbal expression were more likely to be left unexamined.

For example, in one of the integrative cases, a client displayed a recurring pattern of suddenly moving from intense crying into bursts of laughter. This shift was noted by the supervision group, yet their discussion remained at the level of behavioral observation, without further inquiry into the possible defensive or affective functions of this transition. Interestingly, in the IPR interview, the client herself reflected on this dynamic, noting that while she had not been aware of it at the time, she now wondered if the laughter served to devalue her own emotional expression. Had the supervisor facilitated a more reflective exploration of this sequence, the therapist might have been better equipped to help the client recognize and work through the defensive function of her laughter in-session.

Conversely, in one of the dynamic cases, we observed the opposite pattern; the supervision group moved quickly from observation to interpretation. In this therapy process, the client consistently ended her therapy sessions by smiling and saying for example “See you on Friday.” The therapist shared in his interview that the client’s smile evoked a desire to see her again. In the supervision group, they discussed whether the smile was an expression of the client’s attachment to the therapist, or perhaps an attempt by the client to minimize her emotional needs. However, during the therapy the client spoke about a childhood fear that her mother might harm her during the night, and how she had developed a protective ritual of saying “See you tomorrow” before going to bed. During the IPR interview with this client, a previously unnoticed narrative surfaced when the interviewer made the connection between the client’s childhood ritual and her closing words in therapy. The client was visibly moved by this, and it seems reasonable to suggest that these relational scenarios may be understood as a subtext in the client’s smile (Gullestad and Killingmo, 2020). This suggests that, had the supervision group lingered longer with the nuances of the client’s smile, and the emotional and narrative context in which it occurred, they might have uncovered its deeper psychological significance.

These patterns highlight two distinct risks in supervisory practice. First, when supervisors focus solely on what is observable, they may miss the opportunity to help trainees reflect on the affective undercurrents of clinical encounters. Second, when supervision jumps too quickly to interpretation, without attending to the phenomenological details of what is seen or heard, it may obscure the observational foundation upon which accurate emotional understanding depends. These dynamics can be fruitfully understood through the lens of mentalization theory. Lecours and Bouchard (2011) differentiate between various levels of mental processing, including somatization, affect recognition, symbolic representation, and reflective elaboration. Clinical competence involves the capacity to operate across all levels, and to linger productively in the space between them. If supervisors or trainees remain either too anchored in raw observation or are too quick to interpret, the developmental trajectory of reflective functioning may be compromised.

Within the psychoanalytic tradition, these indications in the present study raises questions of vital importance: How effectively do psychoanalysts ensure that their interpretations are grounded in observable data? Is there a tendency to bypass careful observation and prematurely leap to theoretical understanding? Indeed, psychoanalysis has frequently been criticized for privileging subjective interpretation at the expense of empirical rigor, with many critiques pointing out its sparse engagement with systematic observation (Paris, 2017). Eysenck (1985) famously characterized psychoanalysis as more literary art than scientific discipline, arguing that Freud constructed interpretations with little basis in empirical evidence. However, Freud emphasized that theory must be developed from empirical work and not vice versa. He wrote: “For these ideas are not the foundation of the science upon which everything rests. That foundation is observation alone” (Freud, 1914, p. 77). For psychoanalysis to maintain its relevance and continue to influence the broader field of academic psychology, it is essential that both clinicians and researchers take care to ensure that our interpretations are firmly grounded in empirical observations (Hoff et al 2024).

During our work with the nonverbal aspects of psychotherapy, it has been even clearer to us how our ability to verbalize what occurs at a nonverbal level is limited by our verbal language. Verbal frameworks dominate clinical discourse, which may constrain therapists’ and supervisors’ ability to reflect on and teach embodied relational skills. This may also contribute to explain our findings, that the supervisors did not dwell on their clinical observations of nonverbal cues. Moreover, this may help clarify why the supervisors appeared to believe that they worked more intentionally with smiles and laughter than they actually did. However, our immersion in this material has shown that it is possible to cultivate a more refined vocabulary for verbalizing the nonverbal. Hence, we believe that with deliberate attention and practice, clinicians can develop more sophisticated and nuanced ways of thinking about and intervene based on nonverbal communication in the therapeutic relationship, one that strengthens both therapeutic effectiveness and clinical education.

### Strengths, limitations, and further research

A key strength of this study lies in its nuanced, multimodal methodology, including the perspectives of all three central participants in psychotherapy training—the therapist, the client, and the supervisor. In particular, the use of Interpersonal Process Recall (IPR) interviews (Elliot, 1986) produced rich, in-depth material, offering valuable insights into how participants experienced smiles and laughter in the context of psychotherapy. This aligns with recent recommendations by Hill et al. (2025) for more detailed explorations of nonverbal phenomena. The design enabled the capture of subtle interactional dynamics that might have been overlooked using more rigid or standardized methods.

However, the study also has clear limitations. The sample size was small, and some data were missing. We did not include standardized measures of perceived alliance, therapy outcome, or client satisfaction—factors that could have enriched our analysis, particularly regarding client and training outcomes. Exploring participants’ attachment styles might also have provided a broader relational context. In addition, isolating smiles and laughter can be seen as reductionistic, given that these expressions are embedded in a broader stream of nonverbal communication, including body posture, gestures, and prosody. A further limitation is that all participants were of Caucasian origin. As emotions are to a large extent socially constructed and culturally shaped, this homogeneity limits the generalizability of the findings across diverse cultural contexts. Finally, because the supervisors in our cases did not intentionally focus on smiles and laughter in their guidance, we were unable to examine how this competence might be developed when it is explicitly addressed. Future research could fruitfully explore training contexts where nonverbal expression is deliberately targeted and reflected upon in supervision.

## Conclusion

Through a nuanced account of how smiles and laughter were expressed, experienced, and worked with in five training therapies, this study challenges simplified notions of nonverbal synchrony. Instead, it highlights the importance of embodied emotional regulation. Moreover, the study makes explicit how clinical practice unfolds on two interrelated levels: The observable, external expressions that can be seen or heard, and the underlying emotional dynamics that must be inferred and interpreted. It further reminds us that psychoanalytic and more generally psychotherapeutic clinical work must remain grounded in careful empirical observation. At the same time, the study reveals how difficult it can be for clinicians to articulate what they observe at the nonverbal level. The subtlety of bodily expressions, combined with the lack of a rich verbal language for describing them, may constrain reflection and dialogue. Developing a more precise vocabulary for the nonverbal could therefore deepen clinical understanding and support the integration of these often-overlooked dimensions into psychotherapy training and supervision.

## Data Availability

The raw data is not readily available as it consists of sensitive clinical material from naturalistic psychotherapy and supervision sessions. However, a detailed description of the coding framework and the analytical structure can be provided upon request. Requests to access the datasets should be directed to cechoff@gmail.com.

## References

[ref1] Archibald M. M. (2016). Investigator triangulation: a collaborative strategy with potential for mixed methods research. J. Mixed Methods Res. 10, 228–250. doi: 10.1177/1558689815570092

[ref2] Atzil-Slonim D. Soma C. S. Zhang X. Adar Paz A. Imel Z. E. (2023). Facilitating dyadic synchrony in psychotherapy sessions: systematic review and meta-analysis. Psychother. Res. 33, 898–917. doi: 10.1080/10503307.2023.2191803 37001119

[ref3] Bänninger-Huber E. Salvenauer S. (2022). Different types of laughter and their effects on emotion regulation in psychotherapy. Curr. Psychol. doi: 10.1007/s12144-022-03485-1

[ref4] Bar-Kalifa E. Goren O. Gilboa-Schechtman E. Wolff M. Rafael D. Heimann S. . (2023). Clients’ emotional experience as a dynamic context for client-therapist physiological synchrony. J. Consult. Clin. Psychol. 91, 367–380. doi: 10.1037/ccp0000811, PMID: 37104801

[ref5] Barrett L. F. Russel J. A. (2015). The psychological construction of emotion. New York: The Guilford Press.

[ref6] Bedi R. P. Davis M. D. Williams M. (2005). Critical incidents in the formation of the therapeutic alliance from the client’s perspective. Psychother. (Chic.). 42, 311–323. doi: 10.1037/0033-3204.42.3.311

[ref7] Beebe B. Jaffe J. Markese S. Buck K. Chen H. Cohen P. . (2010). The origins of 12-month attachment: a microanalysis of 4-month mother-infant interaction. Attach Hum. Dev. 12, 3–141. doi: 10.1080/14616730903338919 20390524 PMC3763737

[ref8] Beebe B. Lachmann F. M. (2002). Infant research and adult treatment: Co-constructing interactions. London: The Analytic Press.

[ref9] Benecke C. Krause R. (2005). Initial affective facial behavior and outcome satisfaction in the psychotherapy of patients with panic disorder. Z. Psychosom. Med. Psychother. 51, 346–359. doi: 10.13109/zptm.2005.51.4.346 16402333

[ref10] Bion W. R. (1962). Learning from experience. London: Karnac Books.

[ref11] Braun V. Clarke V. (2006). Using thematic analysis in psychology. Qual. Res. Psychol. 3, 77–101. doi: 10.1191/1478088706qp063oa

[ref12] Braun V. Clarke V. (2013). Successful qualitative research: A practical guide for beginners. London: Sage.

[ref13] Braun V. Clarke V. (2019). Reflecting on reflexive thematic analysis. Qual. Res. Sport Exerc. Health 11, 589–597. doi: 10.1080/2159676X.2019.1628806

[ref14] Braun V. Clarke V. (2023). Toward good practice in thematic analysis: avoiding common problems and be(com)ing a knowing researcher. Int. J. Transgender Health 24, 1–6. doi: 10.1080/26895269.2022.2129597, PMID: 36713144 PMC9879167

[ref15] Canestrari C. Dionigi A. (2018). Laughter in cognitive behavioural therapy: a conversational and sequential overview. Discourse Stud. 20, 323–339. doi: 10.1177/1461445618754426, PMID: 41181373

[ref16] Creswell J. W. Poth C. N. (2017). Qualitative inquiry and research design: Choosing among five approaches. Thousand Oaks: Sage.

[ref17] Darwiche J. de Roten Y. Stern D. J. von Roten Crettaz F. Corboz-Warnery A. Fivaz-Depeursinge E. (2008). Mutual smiling episodes and therapeutic alliance in a therapist–couple discussion task. Swiss J. Psychol. 67, 231–239. doi: 10.1024/1421-0185.67.4.231

[ref18] Deres-Cohen K. Dolev-Amit T. Peysachov G. Ramseyer F. T. Zilcha-Mano S. (2021). Nonverbal synchrony as a marker of alliance ruptures. Psychotherapy 58, 499–509. doi: 10.1037/pst0000384, PMID: 34881925

[ref9001] Dreher M. Mengele U. Krause R. Kämmerer A. (2001). Affective indicators of the psychotherapeutic process: An empirical case study. Psychotherapy Research. 11, 99–117., PMID: 25849880 10.1080/713663855

[ref19] Ekman P. (1992). An argument for basic emotions. Cognit. Emot. 6, 169–200. doi: 10.1080/02699939208411068, PMID: 41180919

[ref20] Ekman P. Friesen W. V. (1982). Felt, false, and miserable smiles. J. Nonverbal Behav. 6, 238–252. doi: 10.1007/BF00987191

[ref21] Elliot R. (1986). “Interpersonal process recall (IPR) as psychotherapy process research method” in The psychotherapeutic process: A research handbook. eds. Greenberg L. Pinsof W. (New york: Guildford Press), 503–526.

[ref22] Eysenck H. J. (1985). Decline and fall of the Freudian empire. New York: Viking Press.

[ref23] Feldman R. Greenbaum C. W. Yirmiya N. Mayes L. C. (1996). Relations between cyclicity and regulation in mother-infant interaction at 3 and 9 months and cognition at 2 years. J. Appl. Dev. Psychol. 17, 347–365. doi: 10.1016/S0193-3973(96)90031-3

[ref24] Finlay L. (2021). Thematic analysis: the ‘good’, the ‘bad’ and the ‘ugly’. Europ. J. Qual. Res. Psychother. 11, 103–116. doi: 10.24377/EJQRP.article3062

[ref25] Freud S. (1914). “On narcissism: an introduction” in The standard edition of the complete psychological works of Sigmund Freud. ed. Strachey J., vol. 14 (London: Hogarth Press), 67–102.

[ref26] Freud S. (1923). The ego and the id (Strachey S.). In Strachey J. (Ed.), The standard edition of the complete psychological works of Sigmund Freud (19, 19–26). London: Hogarth Press.

[ref27] Glenn P. J. (2003). Laughter in interaction. Cambridge: Cambridge University Press.

[ref28] Gregorini C. De Carli P. Parolin L. A. L. Preti E. (2025). Potential role of nonverbal synchrony in psychotherapy: a meta-analysis. Couns. Psychother. Res. 25:e12885. doi: 10.1002/capr.12885

[ref29] Gullestad S. E. Killingmo B. (2020). The theory and practice of psychoanalytic therapy: Listening for the subtext. London: Routledge.

[ref30] Haakana M. (2012). “Laughter in conversation: the case of “fake” laughter” in Emotion in interaction. eds. Peräkylä A. Sorjonen M. L. (Oxford: Oxford University Press), 173–194.

[ref31] Hill C. E. Kivlighan D. M. Shaw S. King S. Alford M. Bhalwani S. . (2025). Crying, laughter, and silence in psychodynamic psychotherapy for anxiously and avoidantly attached clients. Couns. Psychol. Q. 25, 1–23.

[ref32] Hill C. E. Knox S. (2013). “Training and supervision in psychotherapy” in Handbook of psychotherapy and behaviour change. ed. Lambert M. J.. 6th ed (Hoboken: John Wiley & sons), 775–811.

[ref9002] Hoff C. H. (2024). Relational competence in psychotherapy. eds. Gullestad S. E. Stänicke E. Bohleber M. L.. Psychoanalytic Studies of Change, An Integrative Perspective. London: Routledge.

[ref9003] Hoff C. H. Nissen-Lie H. A. Strømme H. (2024a). Learning from embodied tension: A naturalistic multimodal single case study of nonverbal expressions and interactions in a psychotherapy training process. European Journal for Qualitative Research in Psychotherapy. 14, 44–61.

[ref33] Holt E. (2016). Laughter at last: playfulness and laughter in interaction. J. Pragmat. 100, 89–102. doi: 10.1016/j.pragma.2016.04.012

[ref34] Huron D. (2006). Sweet anticipation: Music and the psychology of expectation. Cambridge: The MIT Press.

[ref35] Jennissen S. Huber J. Ditzen B. Dinger U. (2024). Association between nonverbal synchrony, alliance, and outcomes: a multilevel meta-analysis. Psychother. Res. 35, 1213–1228. doi: 10.1080/10503307.2024.2423662 39586005

[ref36] Kabir S. (2010). The notion of eternal return in Milan Kundera’s the unbearable lightness of being. Crossings: ULAB journal of. Engl. Stud. 3, 25–41.

[ref37] Knox S. Hill C. E. (2021). “Training and supervision in psychotherapy: what we know and where we need to go” in Bergin and Garfield's handbook of psychotherapy and behavior change: 50th anniversary edition. eds. Barkham M. Lutz W. Castonguay L. G.. 7th ed (Hoboken: John Wiley & Sons, Inc.), 327–349.

[ref38] Koole S. L. (2009). The psychology of emotion regulation: an integrative review. Cogn. Emot. 23, 4–41. doi: 10.1080/02699930802619031

[ref39] Koole S. L. Tschacher W. (2016). Synchrony in psychotherapy: a review and an integrative framework for the therapeutic alliance. Front. Psychol. 7:862. doi: 10.3389/fpsyg.2016.00862, PMID: 27378968 PMC4907088

[ref40] Kundera M. (1984/1999) in The unbearable lightness of being. ed. Heim M. H. (New York: Faber & Faber).

[ref41] Lecours S. Bouchard M.-A. (2011). Verbal elaboration of distinct affect categories and BDP symptoms. Psychol. Psychother. 84, 26–41. 22903829 10.1111/j.2044-8341.2010.02006.x

[ref42] Lemma A. (2016). Introduction to the practice of psychoanalytic psychotherapy. Chichester: Wiley-Blackwell.

[ref43] Levitt H. McLeod J. Stiles W. B. (2021). “The conceptualization, design, and evaluation of qualitative methods in research on psychotherapy” in Handbook of psychotherapy and behavior change. eds. Barkham M. Lutz W. Castonguay L. G.. 7th ed (Hoboken: John Wiley & Sons), 51–86.

[ref44] Levitt H. M. Motulsky S. L. Wertz F. J. Morrow S. L. Ponterotto J. G. (2017). Recommendations for designing and reviewing qualitative research in psychology: promoting methodological integrity. Qual. Psychol. 4, 2–22. doi: 10.1037/qup0000082

[ref45] Marci C. D. Moran E. K. Orr S. P. (2004). Physiologic evidence for the interpersonal role of laughter during psychotherapy. J. Nerv. Ment. Dis. 192, 689–695. doi: 10.1097/01.nmd.0000142032.04196.63, PMID: 15457112

[ref46] McLeod J. (2010). Case study research in counselling and psychotherapy. London: Sage Publications Ltd.

[ref47] Meekums B. Macaskie J. Kapur J. (2016). Developing skills in counselling and psychotherapy: a scoping review of interpersonal process recall and reflecting team methods in initial therapist training. Br. J. Guid. Couns. 44, 504–515.

[ref48] Norcross J. C. Wampold B. E. (2011). Evidence-based therapy relationships: research conclusions and clinical practice. Psychotherapy 48, 98–102. 21401280 10.1037/a0022161

[ref49] Nyman-Salonen P. Tourunen A. Kykyri V.-L. Kaartinen J. Seikkula J. (2021a). Nonverbal synchrony in couple therapy linked to clients’ well-being and alliance. Front. Psychol. 12:718353. doi: 10.3389/fpsyg.2021.718353 34858258 PMC8631962

[ref50] Nyman-Salonen P. Tourunen A. Kykyri V.-L. Kaartinen J. Seikkula J. (2021b). Studying nonverbal synchrony in couple therapy—observing implicit posture and movement synchrony. Contemp. Fam. Ther. 43, 69–87. doi: 10.1007/s10591-020-09555-5

[ref51] Ogden T. H. (1994). The analytic third: working with intersubjective clinical facts. Int. J. Psychoanal. 75, 3–19. 8005761

[ref52] Paris J. (2017). Is psychoanalysis still relevant to psychiatry? Can. J. Psychiatr. 62, 308–312. doi: 10.1177/0706743717692306, PMID: 28141952 PMC5459228

[ref53] Pomeroy E. Weatherall A. (2014). Responding to client laughter as therapeutic actions in practice. Br. J. Soc. Work. 44, 190–206. doi: 10.1093/bjsw/bcs096

[ref54] Ramseyer F. (2020). Exploring the evolution of nonverbal synchrony in psychotherapy: the idiographic perspective provides a different picture. Psychother. Res. 30, 622–634. doi: 10.1080/10503307.2019.1676932, PMID: 31603387

[ref55] Ramseyer F. Tschacher W. (2011). Nonverbal synchrony in psychotherapy: coordinated body movement reflects relationship quality and outcome. J. Consult. Clin. Psychol. 79, 284–295. doi: 10.1037/a0023419, PMID: 21639608

[ref56] Seikkula J. Karvonen A. Kykyri V. L. Kaartinen J. Penttonen M. (2015). The embodied attunement of therapists and a couple within dialogical psychotherapy: an introduction to the relational mind research project. Fam. Process 54, 703–715. doi: 10.1111/famp.12152, PMID: 25810020

[ref57] Tronick E. Beeghly M. (2011). Infants' meaning-making and the development of mental health problems. Am. Psychol. 66, 107–119. doi: 10.1037/a0021631, PMID: 21142336 PMC3135310

[ref58] Tuckett D. (2005). Does anything go? Towards a framework for the more transparent assessment of psychoanalytic competence. Int. J. Psychoanal. 86, 31–49. doi: 10.1516/R2U5-XJ37-7DFJ-DD18, PMID: 15859220

[ref59] Winnicott D. W. (1960). The theory of the parent-infant relationship. Int. J. Psychoanal. 41, 585–595. 13785877

[ref60] Winnicott D. W. (1965). The maturational processes and the facilitating environment. London: Hogarth Press.

[ref61] Winnicott D. W. (1971). Playing and reality. London: Routledge.

[ref62] Zilcha-Mano S. (2024). How getting in sync is curative: insights gained from research in psychotherapy. Psychol. Rev. 132, 470–487. doi: 10.1037/rev0000471 38358717

