# Correction: The unbearable lightness of laughting: a reflexive thematic analysis of smiles and laughter in five psychotherapy training processes

**DOI:** 10.3389/fpsyg.2025.1756365

**Published:** 2025-12-16

**Authors:** Frontiers Production Office

**Affiliations:** Frontiers Media SA, Lausanne, Switzerland

**Keywords:** containment, nonverbal communication, psychoanalytic interpretations, psychotherapy training, smiles and laughter in psychotherapy, supervision, qualitative analysis

The title of this article was erroneously given as: “The unbearable lightness of laughing: a reflexive thematic analysis of smiles and laughter in five psychotherapy training processes.”

The correct title of the article is “The unbearable lightness of laughting: a reflexive thematic analysis of smiles and laughter in five psychotherapy training processes.”

The original version of this article has been updated.

