# Supplementary material for: The unbearable lightness of laughting: a reflexive thematic analysis of smiles and laughter in five psychotherapy training processes
Source: Front Psychol. 2025 Nov 18;16:1720110. doi: 10.3389/fpsyg.2025.1720110 (PMC12671044; doi:10.3389/fpsyg.2025.1720110)
Supplement: Supplementary file 1 [file Table_1.DOCX]

**Appendix 1**

***Interview with the therapists***

| **The therapy:**  Looking back at the therapy process, what stands out most for you?  Can you give some examples?  Retrospectively, what changes do you think you went through during the process?  Can you describe your immediate reflections of how you learned this?  Thinking back at the process, are there things you feel that you were not able to work with? |
| --- |
| **The supervision:**  Let us look at the relationship to your supervisor. How did you experience that?  What do you think made you experience it like that?  Did the relationship change during the process?  If so, how? Were you at some point surprised by this?  How did the supervisor structure the process?  What do you think of this form of supervision?  Did you want something else from supervision?  Where there some things you missed, or some things you were not able to work with?  Why did you miss that? |
| **Nonverbal communication:**  Thinking back at the process, to what extent do you think you were aware of the nonverbal communication between you and the client?  To what extent do you think you were aware of what you communicated nonverbally to the client?  To what extent do you think you were aware of the client’s body language and nonverbal expressions?  To what extent were you aware of what the client communicated nonverbally to you?  Do you think the client’s nonverbal expressions influenced you as a therapist?  Do you remember if you at any time commented on the client’s body language or nonverbal expressions during the process?  If yes, how did you experience this?  If no, do you remember if you were thinking about this during the process?  If no, do you have any thoughts about why you did not comment on this?  If no, how do you think it would have been for you to comment on the client’s nonverbal expressions?  Do you remember if you at any time talked to the client about the nonverbal interactions between you?  Do you remember if you talked about the client’s nonverbal expressions in supervision?  Do you remember if you talked about possible ways to intervene on the client’s nonverbal behavior in supervision? |
| **The interview and research context**  Do you think that your participation in this research project in any way has affected your training process?  How do you feel that we managed to talk about your experiences during training?  How do you feel about the topics I addressed?  What do you think about my way of interviewing you, regarding your possibilities to describe how the training process was for you?  I do not have any more questions. Is there something you want to add before we finish? |

***Interview with the clients***

| **The current situation**  Could you say something about what made you apply for psychotherapy? How were you doing at the time?  Could you say something about how you are doing now, when it comes to your relationship with yourself, other people, and your work?  If you think back at you situation at the beginning of your therapy, and compare it with how you are doing now, what would you say? |
| --- |
| **Outcome**  Thinking back at the treatment you had; do you think it has contributed to the changes you have described?  When you started the treatment, you felt that your problem was…..Do you believe that you during the treatment changed your perspective on your problem?  What was helpful in the treatment? (Something you realized, some experiences during the process, some things the therapist said or did?)  Something you said or did?  Do some specific memories come to your mind?  Were there any specific topics you felt were particularly important to talk about?  What was it about you that made the therapy useful?  Was the treatment different than you expected?  Thinking back at the therapy now, is there anything you feel that you were not able to work with? |
| **Relationship with the therapist**  Let us take a closer look at your relationship with the therapist. How did you experience that?  What stands out when you think about her?  Do you feel that you became attached to her?  Did you feel that she could understand you?  Did you feel that she considered what you expressed or needed?  Did you feel safe?  How did the fact that she was a student affect you?  Were you concerned about her age?  Did you have confidence in her competence as a therapist?  Do you think she changed during the process?  Did your relationship change during the process?  If so, how?  Were you surprised by this change?  Were there things you missed during the therapy?  If yes, what do you think made you miss this?  How free do you think you felt in letting her know, if there was anything you wanted to be different?  In sum, how would you describe your relationship with your therapist? |
| **Nonverbal communication**  Thinking back at the process, to what extent do you think you were aware of the nonverbal communication between you and the therapist?  To what extent do you think you were aware of what you communicated nonverbally to the therapist?  To what extent were you aware of the therapist’s body language and nonverbal communication?  To what extent were you aware of what the therapist communicated nonverbally to you?  Do you think that the therapist’s nonverbal expressions influenced you?  Do you remember if the therapist at any time commented on your body language or your nonverbal expressions?  If yes, how did you experience this?  If no, do you remember if you were thinking about this during the process?  If no, how do you think it would have been if she commented on it?  Do you remember if the therapist at any time talked about the nonverbal interactions between you? |
| **The interview and research context**  Do you think that your participation in this research project in any way has affected the treatment?  How do you feel that we managed to talk about your experiences in therapy?  How do you feel about the topics I addressed?  What do you think about my way of interviewing you, regarding your possibilities to describe how the therapy was for you?  I do not have any more questions. Is there something you want to add before we finish? |

***Interview with the supervisors***

| **The process**  Looking back at the supervision process, what stands out most for you?  Can you give some examples?  What are your thoughts on how the therapist developed during the process?  What are your immediate reflections on how he/she learned this? |
| --- |
| **Relationship with the therapist**  Let us take a closer look at your relationship with the student therapist. How did you experience that?  What do you think contributed to your experiences?  Did the relationship change during the process? If so, did that surprise you?  Thinking back at the process, do you think there were some things that you were not able to work with?  If so, what do you think may have caused that? |
| **Nonverbal communication**  Thinking back at the process, to what extent do you think you were aware of the nonverbal communication between the therapist and the client?  To what extent were you aware of what the client communicated nonverbally to the therapist?  Do you think the client’s nonverbal expressions affected the therapist?  Do you remember talking in supervision about the client’s nonverbal expressions and/or the nonverbal interaction  between the client and the therapist? If so, how did you experience that?  In supervision, did you talk about whether the therapist should comment in the client’s nonverbal expressions?  In supervision, did you talk about how the client in her clinical interventions may be informed by the client’s  nonverbal expressions?  To what extent were you aware of the therapist’s nonverbal expressions?  To what extent were you aware of the therapist’s nonverbal interaction with the client?  Do you remember if you, in supervision, commented the therapist’s nonverbal expressions or way of being?  If so, how did you experience that? If no, do you remember thinking about it? If no, do you have any thoughts about why you did not do it? How do you think it would have been for you to do it?  Being a supervisor, to what extent do you focus on nonverbal relational skills?  Do you have any thoughts about interventions targeting clients’ nonverbal cues? |
| **The interview and research context**  Do you think that your participation in this research project in any way has affected the treatment?  How do you feel that we managed to talk about your experiences in supervision?  How do you feel about the topics I addressed?  What do you think about my way of interviewing you, regarding your possibilities to describe how the therapy was for you?  I do not have any more questions. Is there something you want to add before we finish? |
